# Community health workers’ involvement in the prevention and control of non-communicable diseases in Wakiso District, Uganda

**DOI:** 10.1186/s12992-020-00653-5

**Published:** 2021-01-07

**Authors:** David Musoke, Edwinah Atusingwize, Deborah Ikhile, Sarah Nalinya, Charles Ssemugabo, Grace Biyinzika Lubega, Damilola Omodara, Rawlance Ndejjo, Linda Gibson

**Affiliations:** 1 Department of Disease Control and Environmental Health, School of Public Health, College of Health Sciences, Makerere University, Kampala, Uganda; 2 School of Social Sciences, Nottingham Trent University, Nottingham, UK

**Keywords:** Community health workers, Non-communicable diseases, Risk factors, Knowledge, Attitudes, Practices, Community perceptions, Uganda

## Abstract

**Background:**

Community health workers (CHWs) are an important cadre of the global health workforce as they are involved in providing health services at the community level. However, evidence on the role of CHWs in delivering interventions for non-communicable diseases (NCDs) in Uganda is limited. This study, therefore, assessed the involvement of CHWs in the prevention and control of NCDs in Wakiso District, Uganda with a focus on their knowledge, attitudes and practices, as well as community perceptions.

**Methods:**

A cross-sectional study using mixed methods was conducted which involved a structured questionnaire among 485 CHWs, and 6 focus group discussions (FGDs) among community members. The study assessed knowledge, perceptions including the importance of the various risk factors, and the current involvement of CHWs in NCDs, including the challenges they faced. Quantitative data were analysed in STATA version 13.0 while thematic analysis was used for the qualitative data.

**Results:**

The majority of CHWs (75.3%) correctly defined what NCDs are. Among CHWs who knew examples of NCDs (87.4%), the majority mentioned high blood pressure (77.1%), diabetes (73.4%) and cancer (63.0%). Many CHWs said that healthy diet (86.2%), physical activity (77.7%), avoiding smoking/tobacco use (70.9%), and limiting alcohol consumption (63.7%) were very important to prevent NCDs. Although more than half of the CHWs (63.1%) reported being involved in NCDs activities, only 20.9 and 20.6% had participated in community mobilisation and referral of patients respectively. The majority of CHWs (80.1%) who were involved in NCDs prevention and control reported challenges including inadequate knowledge (58.4%), lack of training (37.6%), and negative community perception towards NCDs (35.1%). From the FGDs, community members were concerned that CHWs did not have enough training on NCDs hence lacked enough information. Therefore, the community did not have much confidence in them regarding NCDs, hence rarely consulted them concerning these diseases.

**Conclusions:**

Despite CHWs having some knowledge on NCDs and their risk factors, their involvement in the prevention and control of the diseases was low. Through enhanced training and community engagement, CHWs can contribute to the prevention and control of NCDs, including health education and community mobilisation.

**Supplementary Information:**

The online version contains supplementary material available at 10.1186/s12992-020-00653-5.

## Introduction

The increased burden of non-communicable diseases (NCDs) and related mortality is a major public health issue especially among low-and middle-income countries (LMICs) [1, 2]. About 70% of all deaths globally are caused by NCDs, of which 87% occur in LMICs [3]. In Uganda, the occurrence of NCDs has been increasing steadily in recent years. For instance, data from the outpatient department at health facilities in the country showed that between 2012 and 2016, hypertension increased from 60,000 to 85,000 cases, while diabetes cases rose from 30,000 to 32,000, an increase of 42 and 7% respectively [4]. A national risk factor survey in 2014 showed that one in 10 persons aged 40–69 years had a 10-year cardiovascular disease (CVD) risk ≥30%, or existing CVD [5]. In addition, only 10% of women aged 30–49 years had been screened for cervical cancer, the leading cause of cancer deaths in Uganda [4, 5]. Similarly, the prevalence of hypertension in the Ugandan population is high, yet most people with the disease are not aware of their status [5–7].

Community health workers (CHWs) are acknowledged as an important part of the public health workforce globally and are mobilised to provide basic health care at the community level [8]. CHWs are trained individuals that assist in providing community empowerment, administering basic health services, and linking local communities with health facilities to reduce morbidity and mortality [9]. However, their role and contributions in many countries, including Uganda, are largely limited to maternal and child health as well as prevention and treatment of infectious diseases [10]. CHWs are often trained to fill the provider gaps in health systems and health care delivery, especially in LMICs, due to the shortage of human resources. In Uganda and other LMICs, addressing the recognised major NCDs such as CVD, cancer, diabetes and chronic respiratory diseases is important at the primary care level considering the rising burden of morbidity and mortality they are causing [3, 11]. Health programmes and action plans for NCDs are therefore being strengthened for primary prevention, and CHWs have the potential to offer support in the provision of primary health care services [12].

To address the gaps in human resources for health regarding NCDs, mobilisation of CHWs seems promising [13, 14]. Evidence suggests varied roles and relevance of CHWs in the management of NCDs [15]. These roles include patient education and care, provision of social support, and acting as a liaison with the healthcare system [16]. For example, in a diabetes intervention, CHWs assisted in monitoring blood glucose, blood pressure and potential complications, and provided social support to patients as well as their families [17]. While CHWs execute extensive community mobilisation, empowerment for disease prevention and health promotion for communicable diseases as well as maternal and child health [18, 19], there is limited simultaneous action on NCDs in communities. Therefore, there is a need to further understand the role of CHWs in the prevention and management of NCDs, especially in resource-poor settings.

Despite other countries having established the role of CHWs in NCDs prevention and control [20–22], evidence about the role of CHWs in delivering primary prevention interventions for NCDs in Uganda is limited [23]. Moreover, evidence suggests that CHWs are interested in deliveringcardiovascular disease prevention services within their communities [24] and thus it is crucial to understand their level of knowledge, attitudes and perceived role in addressingNCDs as part of the feasibility of involving them in the control of these diseases [25]. In addition, information on knowledge and attitudes of CHWs regarding NCDs would provide insights into the competencies and training needs for them to perform their tasks effectively [10]. This study, therefore, assessed the involvement of CHWs in the prevention and control of NCDs in Wakiso District, Uganda with a focus on their knowledge, attitudes and practices, as well as community perceptions. Understanding the perceptions of the community on the level of involvement of CHWs in NCDs is crucial given they are the main recipients of their services. Indeed, community members are key stakeholders regarding the work of CHWs [26], therefore, their views concerning the services offered by the health volunteers is important to inform future decisions.

## Methods

### Study design and data collection

The study was cross-sectional and involved mixed methods of data collection, with the quantitative and qualitative data collected concurrently. The quantitative component of the study involved administering a structured questionnaire in the local language (*Luganda*) by Research Assistants among CHWs to establish their knowledge, attitudes and involvement in the prevention and control of NCDs, and had four sections. The first section comprised of questions on demographic characteristics of participants such as age, gender, level of education, and whether they were involved in integrated community case management (iCCM) of childhood illnesses. The second section assessed knowledge of CHWs on NCDs, including whether they could correctly define NCDs or knew examples of such diseases, as well as if they thought the illnesses could be prevented and/or treated. The third section explored perceptions of CHWs towards NCDs particularly how they rated the importance of the following risks factors: smoking/tobacco use, alcohol consumption, physical inactivity, and unhealthy diet using a Likert scale (very important, important, fairly important, not important). Attitudes towards NCDs were determined using a Likert scale (agree, neither agree nor disagree, disagree). This section also explored the potential role that CHWs can play in the prevention and control of NCDs in the community. The fourth section assessed the current involvement of CHWs in the prevention and control of NCDs, including any challenges they faced while doing so. CHWs practices such as community mobilisation; early detection; screening; referral of patients to health facilities; and supporting adherence to treatment were examined. The study assessed the following challenges among CHWs regarding their involvement in the prevention and control of NCDs: low knowledge; lack of training on NCDs; poor community perception towards NCDs; and lack of support from health workers.

For the qualitative component, focus group discussions (FGDs) were conducted among community members to obtain their views on the involvement of CHWs in the prevention and control of NCDs. The major themes explored in the FGDs were potential involvement of CHWs in NCDs, the involvement of CHWs in NCDs prevention and control at the time, challenges faced / could be faced by community members while seeking services from CHWs on NCDs, and how the involvement of CHWs in NCDs could be improved. These FGDs were also conducted in the local language and audio recorded in a field office located in the study area.

### Study area, context and sampling

The study was conducted in Busiro South health sub-district in Wakiso district, which is comprised of Kajjansi, Kasanje and Katabi town councils, and Bussi sub-county. This health sub-district was involved in the research due to having both rural and peri-urban populations. In addition, Busiro South neighbours Entebbe Municipality (a predominantly urban setting) and other rural areas in the district. Given that many health sub-districts across the country are composed of both rural and peri-urban communities, the findings from this study site can be generalisable to similar settings. According to the 2014 national census, the four administrative units had the following population: Kajjansi (93,238), Kasanje (30,276), Katabi (104,335), and Bussi (16,460) [27]. The population in the area is involved in various economic activities including agriculture, business, stone quarrying, sand mining and fishing. Each of the villages in the health sub-district is mandated to have 4 CHWs among whom 2 are particularly involved in treating childhood illnesses of malaria, diarrhoea and pneumonia under iCCM in addition to other roles such as health education, community mobilisation and health promotion. Although the health sub-district has over 500 CHWs, all the 485 CHWs available in the community during data collection were involved in the study. A total of 6 FGDs were conducted among the community as follows: 1 adult male; 2 adult female; 1 adult mixed (male and female); and 2 mixed youth (male and female). The youth involved in the study were aged between 18 and 30 years. Separate FGDs for youth were held as sometimes they are not open to discussion when taking part in sessions involving their elders. Three randomly selected villages in the health sub-district were involved in the FGDs, with each group comprising of at least 8 and at most 12 members. The selection of FGD participants was done purposively by community mobilisers in consultation with the local leaders based on those who were deemed knowledgeable about the work of CHWs. The 6 FGDs were sufficient to reach data saturation based on the objectives of the study.

### Data analysis

Quantitative data were entered in EpiData version 3.02 and univariate analysis conducted in STATA version 13.0 including generation of frequencies and percentages. Qualitatively, the recordings were labelled and transcribed verbatim in the local language and later translated to English. The transcripts were proof-read by two researchers experienced in qualitative research to familiarise themselves with the data and harmonise any incoherence. Thematic analysis was manually conducted where sections of texts that discussed similar issues were highlighted and linked together to develop codes and themes [28, 29]. The development of themes was also informed by the results obtained from the quantitative data [30] in line with the study objectives.

### Ethical considerations

The study received ethical approval from Makerere University School of Public Health Higher Degrees, Research, and Ethics Committee. The study also had clearance from the Uganda National Council for Science and Technology (UNCST). Written informed consent was obtained from all participants after explaining to them the purpose of the study and stressing that their involvement was voluntary.

## Results

### Demographic characteristics of CHWs

The majority of CHWs were females 74.8% (363/485) and married 72.4% (351/485). On average, the CHWs were aged 42.4 years (SD ± 11.6) and had served in this role for 7.6 years (SD ± 4.7). More than a half of the CHWs had secondary (ordinary) level as their highest education 53.4% (259/485) and were involved in iCCM 56.9% (276/485) (Table 1).

**Table 1:** Demographic characteristics of CHWs

Characteristic	Categories	Frequency (***n*** = 485)	Percentage (%)
Sub-county / town council	Kajjansi	262	54.0
	Kasanje	85	17.5
	Katabi	66	13.6
	Bussi	72	14.9
Age (years)	(Mean = 42.4, Standard deviation ±11.6)		
	20–29	71	14.6
	30–39	137	28.3
	40–49	135	27.8
	50–59	99	20.4
	> 59	43	8.9
Gender	Male	122	25.2
	Female	363	74.8
Religion	Catholic	216	44.5
	Anglican	165	34.0
	Muslim	32	6.6
	Pentecostal	53	10.9
	Seventh-Day Adventists	17	3.5
	Other	2	0.4
Highest education level	None	1	0.2
	Pre-primary	2	0.4
	Primary	130	26.8
	Ordinary secondary	259	53.4
	Advanced secondary	42	8.7
	Tertiary	51	10.5
Marital status	Married	351	72.4
	Single	55	11.3
	Widowed	37	7.6
	Divorced	42	8.7
Involved in iCCM	Yes	276	56.9
	No	209	43.1
Length of service as a CHW (years)	(Mean = 7.6, standard deviation ±4.7)		
	0–10	365	75.3
	> 10	120	24.7

### Knowledge of CHWs about NCDs

The majority of CHWs 75.3% (365/485) correctly defined what NCDs are. Among CHWs who knew examples of NCDs 87.4% (424/485), the majority mentioned high blood pressure 77.1% (327/424), diabetes 73.4% (311/424) and cancer 63.0% (267/424). Most CHWs said that NCDs were preventable 71.8% (348/485) and curable 89.9% (436/485). CHWs were generally aware of risk factors for NCDs ranging from environmental pollution to lifestyle practices. For instance, they acknowledged that ways of preventing NCDs include healthy diet 60.1% (209/348), reducing environmental exposures 36.8% (128/348), and not smoking / using tobacco 31.9% (111/348) (Table 2).

**Table 2:** Knowledge of CHWs about NCDs

Characteristic	Category	Frequency (***n*** = 485)	Percentage (%)
Defined NCDs correctly	Yes	365	75.3
	No	120	24.7
Knew examples of NCDs	Yes	424	87.4
	No	61	12.6
Examples of NCDs (*n* = 424)	Cancer	267	63.0
	Diabetes	311	73.4
	High blood pressure	327	77.1
	Cardiovascular diseases	52	12.3
	Mental health conditions	55	13.0
	Chronic respiratory diseases	24	5.7
	Sickle cell anaemia	73	17.2
	Ulcers	75	17.7
NCDs are preventable	Yes	348	71.8
	No	137	28.3
Ways of preventing NCDs (*n* = 348)	Not smoking / using tobacco	111	31.9
	Regular physical activity	97	27.9
	Healthy diet	209	60.1
	Limiting alcohol consumption	56	16.1
	Reducing environmental exposures	128	36.8
	Others	25	7.2
NCDs are curable	Yes	436	89.9
	No	49	10.1

### CHWs attitudes and potential role in NCDs prevention and control

The majority of CHWs 73.6% (357/485) agreed that NCDs were common among people in their community. In addition, many CHWs said that a healthy diet 86.2% (418/485), physical activity 77.7% (377/485), avoiding smoking / using tobacco 70.9% (344/485) and limiting alcohol consumption 63.7% (309/485) were very important to prevent NCDs. In relation to their potential contribution, nearly all CHWs 94.4% (458/485) agreed that they had a role they could play in the prevention and control of NCDs in their communities. The role included health education 96.3% (441/458), screening and early detection 19.2% (88/458), as well as referral of patients to health facilities 37.0% (169/458) (Table 3).

**Table 3:** CHWs attitudes and potential role in the prevention and control of NCDs

Characteristic	Category	Frequency (***n*** = 485)	Percentage (%)
**Perceptions towards NCDs**			
NCDs are common among people in the community	Agree	357	73.6
	Neither agree nor disagree	30	6.2
	Disagree	98	20.2
**Importance of NCDs risk factors**			
Avoiding smoking / using tobacco	Very important	344	70.9
	Important	63	13.0
	Fairly important	62	12.8
	Not important	16	3.3
Limiting alcohol use	Very important	309	63.7
	Important	69	14.2
	Fairly important	87	17.9
	Not important	20	4.1
Physical activity	Very important	377	77.7
	Important	61	12.6
	Fairly important	37	7.6
	Not important	10	2.1
Healthy diet	Very important	418	86.2
	Important	47	9.7
	Fairly important	16	3.3
	Not important	4	0.8
**Role in prevention and control of NCDs**			
CHWs have a role to play in prevention and control of NCDs	Agree	458	94.4
	Neither agree nor disagree	6	1.2
	Disagree	21	4.3
Role CHWs could play in the prevention and control of NCDs (*n* = 458)	Health education	441	96.3
	Community mobilisation	140	30.6
	Screening and early detection	88	19.2
	Referral	169	37.0
	Supporting adherence to treatment	15	3.3

### Involvement of CHWs in the prevention and control of NCDs

More than half of the CHWs 63.1% (306/485) reported being involved in NCD prevention and control activities including community mobilisation 20.9% (64/306), referral 20.6% (63/306), screening and detection 12.7% (39/306), and supporting adherence to treatment 2.0% (6/306). Similarly, community members who participated in the FGDs confirmed that CHWs were a key component of the workforce for health service delivery, including in the prevention and control of NCDs. The FGD participants said that CHWs were mainly involved in mobilising communities to go for cancer screening and referring those with NCDs symptoms to health facilities.

*“They* [CHWs] *mobilise us whenever there is an important health event such as cancer screening. CHWs are usually approached by health workers who ask them to mobilise us to go to a central location in the community such as the sub-county headquarters for such an activity. The CHWs try their best to communicate to all community members to take part.”* Participant 6, Female FGD 2

The majority of CHWs 80.1% (245/306) who were involved in NCDs prevention and control reported challenges including inadequate knowledge on NCDs 58.4% (143/245), lack of training on NCDs 37.6% (92/245), and negative community perception towards NCDs 35.1% (86/245). Only 69.5% (337/485) of the CHWs said they had received some training on NCDs prevention and control which was mainly from non-governmental organisations (NGOs) / other partners 87.2% (294/337) (Table 4).

**Table 4:** Practices of CHWs in the prevention and control of NCDs

Characteristic	Categories	Frequency (***n*** = 485)	Percentage (%)
Involvement in NCDs prevention and control	Yes	306	63.1
	No	179	36.9
NCD prevention and control activities involved (*n* = 306)	Community mobilisation	64	20.9
	Screening and early detection	39	12.7
	Referral	63	20.6
	Supporting adherence to treatment	6	2.0
Examples of NCDs CHWs were involved in their prevention and control (*n* = 306)	Cancer	143	46.7
	Diabetes	171	55.9
	High blood pressure	195	63.7
	Cardiovascular diseases	19	6.2
	Others	35	11.4
Faced challenges while involved in the prevention and control of NCDs (*n* = 306)	Yes	245	80.1
	No	61	19.9
Challenges involved in the prevention or control of NCDs (*n* = 245)	Low knowledge	143	58.4
	Lack of training on NCDs	92	37.6
	Negative community perception towards NCDs	86	35.1
	Lack of support from health workers	29	11.8
	Others	13	5.3
Received training in NCD prevention and control	Yes	337	69.5
	No	148	30.5
Organisers of training (*n* = 337)	Ministry of Health	33	9.8
	District Health Office	35	10.4
	NGO / other partners	294	87.2

The challenges faced by the CHWs were also emphasised by some community members. Although FGD participants agreed that CHWs were to an extent involved in the prevention and control of NCDs, most of them said that health education regarding NCDs was limited as their focus was on other health concerns such as water, sanitation and hygiene.

*“CHWs have had limited participation in health education regarding prevention and control of NCDs in our village. They mainly health educate the community on general cleanliness and hygiene but have not done much sensitisation on NCDs such as cancer and diabetes.”* Participant 4, Youth FGD 1

Community members were also concerned that CHWs did not have enough training on NCDs hence lacked enough information about these diseases. Therefore, the community did not have much confidence in CHWs, and rarely consulted them about NCDs. The participants added that they did not get much help or information whenever they consulted CHWs about NCDs and hence further lost belief in them.

*“It is difficult to seek advice about NCDs from CHWs because they do not know a lot about those diseases as I think they have not been adequately trained on them. We know that they have been trained extensively on communicable diseases, so you don’t want to go asking them about NCDs that they do not know much about.*” Participant 8, Male FGD 2

There were also some concerns among participants regarding confidentiality. Some FGD participants thought that CHWs would breach their confidentially if consulted on NCDs, and thus many community members did not feel comfortable seeking advice from them. Since there is a stigma associated with some NCDs such as cancer, the participants feared that CHWs would disclose this information to their relatives and friends within the area.

*“The challenge that we may find is that some CHWs do not always keep secrets yet some of these diseases* [NCDs] *would need to be kept confidential. You may tell your personal condition to a CHW but then you find the entire village knows about it. This may prevent us from going to them to seek support on NCDs.”* Participant 3, Female FGD 1

## Discussion

Our study assessed the involvement of CHWs in NCDs prevention and control, and community perceptions about their role in Wakiso District, Uganda. CHWs are integral to addressing accessibility challenges and the human resource shortfall in health service delivery in Uganda and elsewhere. Although the study findings revealed that most CHWs were knowledgeable about the different types of NCDs and preventive measures through lifestyle modification, their role in the prevention and management of these diseases was minimal. From the findings, it is evident that CHWs knew that they had a potential role to play in NCDs prevention and control. However, this potential, as well as existing roles of CHWs, were impeded by health system challenges including lack of training, inadequate support from health workers, and community members’ perceptions about their capability to deliver NCDs services. Our findings reinforce existing literature on the importance of CHWs’ contribution in addressing the growing burden of NCDs [15, 24, 31]. In addition, our study highlights areas of possible interventions to enhance their capacity to contribute towards NCDs prevention and management, including through training.

The majority of CHWs were aware of the common NCDs, which is consistent with a recent study conducted in Eastern Uganda where CHWs highlighted that diabetes, cancer and high blood pressure were common NCDs [23]. The high level of awareness associated with high blood pressure in this study corresponds with the high prevalence of this disease in Uganda [4, 7, 31], specifically in the central region [7] where our study was conducted. Although there is a recognition of mental health conditions as major NCDs in Uganda [32], in our study, only a few CHWs mentioned them. A possible reason for the low recognition of mental health disorders as NCDs by CHWs is their association with spiritual causes, hence may not be regarded as medical conditions [33]. Similar to our study findings, Jones et al. [34] highlighted that the awareness of chronic respiratory diseases is still low among health workers in Uganda. In addition, conditions such as sickle cell anaemia and ulcers are still not commonly recognised as NCDs in Uganda as established in our study and elsewhere [23]. Future interventions on NCDs among CHWs may therefore provide emphasis on the less known ones such as chronic respiratory diseases and mental health conditions to increase awareness about them. Among the CHWs involved in our study, 89.9% thought NCDs were curable. Although some NCDs such as cancer can be cured if detected early, most of these diseases are chronic hence require long-term management [35]. This general misconception among the CHWs may be attributed to the limited training they had received on NCDs as established in the study.

Not only were the CHWs aware of the different NCDs, but the majority also knew about how to prevent them, reflecting fundamental knowledge about these diseases. Many CHWs accurately indicated major lifestyle preventive measures such as healthy diet, avoiding smoking / using tobacco products, regular physical activity, and limiting alcohol consumption as ways of preventing NCDs. These lifestyle preventive measures can be enhanced in the population through health promotion which CHWs can contribute to. Importantly, the attitudes of CHWs around the importance of NCDs risk factors buttress the point that not only were they aware of NCDs but also had nuanced knowledge about these diseases. Specifically, the lifestyle-related risk factors were considered very important by the majority of CHWs. These risk factors, which were historically associated with high-resource settings, are now becoming pervasive even among low-resource African communities [36] such as Uganda as a result of urbanisation, demographic transition and epidemiological transition [37]. Indeed, the increase in these risk factors has contributed to the high prevalence of NCDs in the country hence the need to explore interventions to address them.

The attitudes of CHWs in our study further reflected the current practices in the country where the majority of the population are not sufficiently carrying out measures to prevent NCDs. For example, a high rate of alcohol consumption and low intake of fruits and vegetables in Uganda have been reported in a related study [38]. It has further been highlighted that low sensitisation around lifestyle risk factors has an impact on the prevalence of NCDs in the country [39]. The knowledge of risk factors for NCDs is an important first step towards preventing these diseases as many of them share risk factors [32, 34]. Therefore, there is a need for increased sensitisation, which can be provided by CHWs, to improve knowledge of NCDs and their risk factors in communities.

Almost all the CHWs perceived themselves as having a role to play in the prevention and control of NCDs. This finding is a strong indication of CHWs’ commitment and willingness to promoting general health and wellbeing at the community level. This agreed with a recent Ugandan study which revealed that CHWs were keen to support with cardiovascular disease prevention [24]. The potential role of CHWs in NCDs prevention and control is similar to their existing responsibility in infectious diseases and maternal / child health which include health education, community mobilisation, referral and supporting adherence to treatment [40, 41]. Indeed, health education and community mobilisation are core roles of CHWs in Uganda [18]. However, there have been concerns in existing literature around overburdening CHWs who are already contributing substantially to the burden of communicable diseases and related health issues [15]. Therefore, the contribution of CHWs to NCDs control needs to be planned with enough support to ensure that they are able to perform optimally without being overburdened particularly in countries where they are volunteers such as Uganda.

Although over half of the CHWs reported being currently involved in NCDs prevention and control, specific activities related to mobilising community members, providing referrals to health facilities, screening and early detection of NCDs, and supporting adherence to treatment was low. The role of CHWs in community mobilisation for NCD services also emerged from the FGDs. A recent community-based train-the-trainer programme reported the successful use of CHWs to promote behavioural change towards the control of chronic respiratory diseases in Uganda [34]. A systematic review showed that CHWs’ role in diabetes management span health education, health promotion, adherence to treatment and providing social support [40]. In addition, a study carried out in South Africa revealed that CHWs could also play an advisory role and support with adherence to treatment [31]. These findings semphasise the potential role of CHWs in NCDs prevention and control in line with their current role for other diseases. Therefore, the current experiences of CHWs in the management of communicable diseases can be leveraged upon in contribution towards addressing the growing burden of NCDs in Uganda at the community level.

Our study identified challenges concerning CHWs’ involvement in NCDs control such as inadequate knowledge, lack of training, lack of support from health workers and negative perceptions of community members towards the diseases. It also emerged from the FGDs that community health education by CHWs on NCDs was minimal probably due to limited knowledge. Similar challenges have been highlighted in relation to the work of CHWs in Uganda [19, 23, 33]. Although over half of the CHWs had received some training on NCDs, the training was mainly provided by NGOs / partners as opposed to the Ministry of Health (MOH) / District Health office hence may not have been part of the mainstream primary health care delivery structure. These challenges also reflected the lack of a structured programme for CHWs involvement in NCDs in the health system in Uganda currently. Perhaps the most crucial challenge that needs to be addressed by key stakeholders, including the MOH and development partners is enhancing the capacity of CHWs in NCDs.

The current focus of CHWs on infectious diseases [31], and inadequate training on NCDs have impacted on the community’s perceptions around CHWs capability to provide appropriate advice on NCDs. As a result, community members reported low confidence and belief in CHWs concerning the management of these diseases. Another concern raised by community members was around confidentiality. Similar concerns regarding confidentiality have been reported in relation to CHWs programmes in other parts of Africa such as South Africa [42] and Swaziland [43], and affect performance and acceptability of their services by community members [42, 44]. Therefore, CHWs providing NCDs prevention and control services requires strong community engagement to ensure that their services are provided in an appropriate and acceptable manner to the population.

A strength of our study was that it involved both quantitative and qualitative data which enabled triangulation of findings. In addition, the study not only obtained information from CHWs but also community members who are the recipients of their services. Another strength of the study was that it involved all CHWs in a health sub-district hence the findings can relate to similar settings in the country.

## Conclusion

Despite CHWs having some knowledge on NCDs and their risk factors, their involvement in the prevention and control of the diseases was limited. Through enhanced training, supervisory support and community engagement, CHWs can contribute to the prevention and control of NCDs, including through health education, community mobilisation, screening and referral of patients to health facilities. While carrying out these roles, CHWs would greatly contribute to primary health care for NCDs at the community level and benefit the broader health system.

## Supplementary Information

**Additional file 1:** Study questionnaire.

## Data Availability

Data from the study are available from the corresponding author on reasonable request.

## Abbreviations

CHW: Community health worker
FGD: Focus group discussion
iCCM: Integrated community case management of childhood illnesses
LMIC: Low- and middle-income country
MOH: Ministry of Health
NCDs: Non-communicable diseases
NGO: Non-governmental organisation
