# Supplementary material for: Community health workers’ involvement in the prevention and control of non-communicable diseases in Wakiso District, Uganda
Source: Global Health. 2021 Jan 7;17:7. doi: 10.1186/s12992-020-00653-5 (PMC7791672; doi:10.1186/s12992-020-00653-5)
Supplement: Supplementary file 1 — Additional file 1: Study questionnaire. [file 12992_2020_653_MOESM1_ESM.pdf]

KNOWLEDGE, ATTITUDES AND PRACTICES OF VILLAGE HEALTH TEAMS ON NON-COMMUNICABLE DISEASES

Demographic / general information

No.	Prompt	Response
1.	Sub county / town council	1 = Ssisa / Kajjansi<br>2 = Kasanje<br>3 = Katabi<br>4 = Bussi
2.	Age ***(provide completed years)***	………………………………
3.	Sex	1 = Male<br>2 = Female
4.	Religion	1 = Catholic<br>2 = Anglican<br>3 = Muslim<br>4 = Pentecostal<br>5 = Seventh Day Adventists (SDA)<br>6 = Other (specify) …………………………………..
5.	What is the highest level of education you attained?	1 = None<br>2 = Pre-primary<br>3 = Primary (P1 to P7)<br>4 = O level (S1 to S4)<br>5 = A level (S5 to S6)<br>6 = Tertiary / university
6.	How long have you worked as a VHT?<br><br>***(Record 0 if less than 1 year. Provide completed years)***	………………………………..
7.	What is your marital status?	1 = Married / cohabiting<br>2 = Single<br>3 = Widowed<br>4 = Divorced / separated
8.	Are you involved in treatment of childhood illnesses?	1 = Yes<br>2 = No

Knowledge on NCDs

No.	Prompt	Response
9.	Do you know what NCDs are?<br>***(Probe to confirm yes response)***	1 = Yes<br>2 = No ***(Define what NCDs are in simple terms)***
10.	Do you know any examples of NCDs?	1 = Yes<br>2 = No ***(Skip to 12)***
11.	Which NCDs are you aware of?<br><br>***(Multiple answers possible)***	1 = Cancer<br>2 = Diabetes<br>3 = High blood pressure<br>4 = Cardiovascular diseases<br>5 = Mental health conditions (such as epilepsy)<br>6 = Chronic respiratory diseases<br>7 = Sickle cell anemia<br>8 = Other (specify) …………………….…………..
12.	Can NCDs be prevented?	1 = Yes<br>2 = No ***(Skip to 14)***
13.	How can NCDs be prevented?<br><br>***(Multiple answers possible)***	1 = Preventing smoking / tobacco use<br>2 = Regular physical activity<br>3 = Proper diet<br>4 = Limiting alcohol consumption<br>5 = Reducing environmental exposures<br>6 = Other (specify) ……………….……………………..
14.	Can NCDs be treated?	1 = Yes<br>2 = No

Attitudes towards NCDs

No.	Prompt	Response
15.	NCDs are common among people in your community.	1 = Agree 2 = Neither agree nor disagree<br>3 = Disagree
16.	How important are these behavioural risk factors in the prevention of NCDs?	
	(a) Avoiding smoking / tobacco use	1 = Very important 2 = Important 3 = Fairly important 4 = Not important
	(b) Limiting alcohol use	1 = Very important 2 = Important 3 = Fairly important 4 = Not important
	(c) Physical activity	1 = Very important 2 = Important 3 = Fairly important 4 = Not important
	(d) Healthy diet	1 = Very important 2 = Important 3 = Fairly important 4 = Not important
17.	VHTs have a role they can play in the prevention and control of NCDs in your community?	1 = Agree 2 = Neither agree nor disagree ***(Skip to 19)***<br>3 = Disagree ***(Skip to 19)***

18.	What role can VHTs play in the prevention and control of NCDs in your community?<br><br>***(Multiple answers possible)***	1. Health education<br>2. Community mobilization for interventions<br>3. Early detection<br>4. Screening<br>5. Referral<br>6. Supporting adherence to treatment<br>7. Other (specify) ……………..……………………

Practices

No.	Prompt	Response
19.	Have you ever been involved in prevention or control of NCDs in your community?	1 = Yes<br>2 = No ***(Skip to 24)***
20.	In what ways have you been involved in prevention or control of NCDs in your community?	1 = Health education<br>2 = Community mobilization for interventions<br>3 = Early detection<br>4 = Screening<br>5 = Referral<br>6 = Supporting adherence to treatment<br>7 = Other (specify) …………………………………..
21.	Which NCDs have you been involved in their prevention or control?<br><br>***(Multiple answers possible)***	1 = Cancer<br>2 = Diabetes<br>3 = High blood pressure<br>4 = Cardiovascular diseases<br>5 = Other (specify) …………………………………..
22.	Have you faced any challenges during your involvement in prevention or control of NCDs?	1 = Yes<br>2 = No ***(Skip to 24)***
23.	What challenges have you faced during your involvement in the prevention or control of NCDs in your community?	1 = Low knowledge<br>2 = Lack of training on NCDs<br>3 = Poor community perception towards NCDs<br>4 = Lack of support from health workers<br>5 = Other (specify) ……………….…………………
24.	Have you ever received any training in NCD prevention or control?	1 = Yes 2 = No ***(End interview)***
25.	Who organised the training (s)?<br><br>***(Multiple answers possible)***	1 = Ministry of Health<br>2 = District health office / health facility<br>3 = Non-governmental Organization<br>4 = University<br>5 = Other (specify) …………………………………..

Thank you very much for your time.

Name of data collector: ……………………………………………………………………………
